# A Case of Contralateral Pneumothorax, Pneumomediastinum, and Pneumopericardium after Dual-Chamber Pacemaker Implantation

**DOI:** 10.1155/2022/4295247

**Published:** 2022-12-03

**Authors:** Wajeeh ur Rehman, Altif Muneeb, Usama Sakhawat, Pranava Ganesh, Nabil Braiteh, Vince Skovira, Waseem Sajjad, Afzal ur Rehman

**Affiliations:** ^1^Department of Internal Medicine, United Health Services Hospitals, Wilson Regional Medical Center, Johnson City, NY, USA; ^2^Department of Cardiology, United Health Services Hospitals, Heart and Vascular Institute, Wilson Regional Medical Center, Johnson City, NY, USA

## Abstract

As permanent pacemaker implantation is increasingly becoming a common practice, it is important to understand potential complications associated with the procedure. We present a 78-year-old Caucasian female who developed contralateral pneumomediastinum, pneumothorax, and pneumopericardium after undergoing implantation of a dual-chamber pacemaker.

## 1. Case Summary

A 79-year-old Caucasian female with significant past medical history of atrial fibrillation not on oral anticoagulation, chronic obstructive pulmonary disease, gastroesophageal reflux disease, hypothyroidism, hyperlipidemia, and congestive heart failure with low ejection fraction presented with severe fatigue and dizziness. According to the patient, she was in a normal state of health one month ago when she started developing shortness of breath on exertion and orthopnea. Over the past week, her symptoms had gotten progressively worse. In addition to these complaints, she also had developed new substernal chest discomfort over the past week. The patient was planning to undergo elective colonoscopy when pre-procedural electrocardiogram (EKG) revealed a complete third-degree heart block. The patient was brought to the emergency department (ED) by emergency medical services (EMS). At the time of presentation in the ED, the patient was feeling dizzy and weak. The patient has also had associated symptoms of palpitations and lightheadedness. The patient denied having any loss of consciousness or syncope.

EKG was repeated, which again confirmed normal sinus rhythm with a third-degree atrioventricular (AV) block with stable ventricular escape rhythm at a ventricular rate of approximately 23 bpm.

On subsequent EKGs, there was found to be changing ectopic ventricular foci; on one EKG, it appeared to be localizing to Left ventricular (LV) basal anteroseptal region; on another, to the basal inferoseptal region.

The patient was admitted to telemetry. Overnight, the patient had a long 10-second AV block without any ventricular escape rhythm associated with syncope. Therefore, the patient underwent transvenous temporary pacemaker insertion via right femoral vein with a rate of 80, output of 10 mg, and sensitivity of 5 mV.

The next morning, the patient underwent implantation of dual-chamber permanent pacemaker with removal of temporary venous pacemaker for complete third-degree heart block [1].

The pacemaker pocket was created by an infraclavicular incision in pectoralis major for Boston Scientific ESSENTIO ELDR, Delaware, USA pacemaker generator.

Access was obtained in the left axillary vein after which the right ventricular pacing lead was placed followed by a right atrial pacing lead using single-plane fluoroscopy. At the time of implantation, the right atrial lead pacing threshold was 0.7 V at 0.5 ms, sensing at 3.5 mV, and impedance of 519 Ω, (Medtronic 4076, Minneapolis, Minnesota, USA). The right ventricular lead pacing threshold was 0.5 V at 0.5 ms, sensing at 11.2 mV, and impedance of 790 Ω (Medtronic 4076).

**Figure 1:**
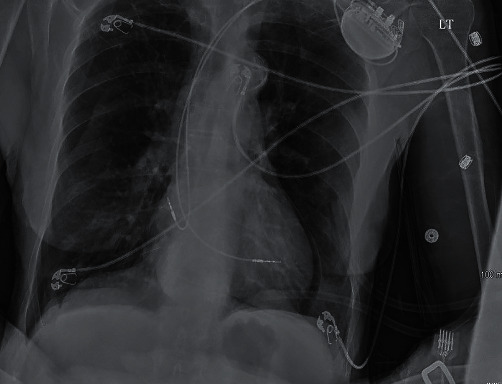
Anteroposterior chest radiograph revealing pneumopericardium.

**Figure 2:**
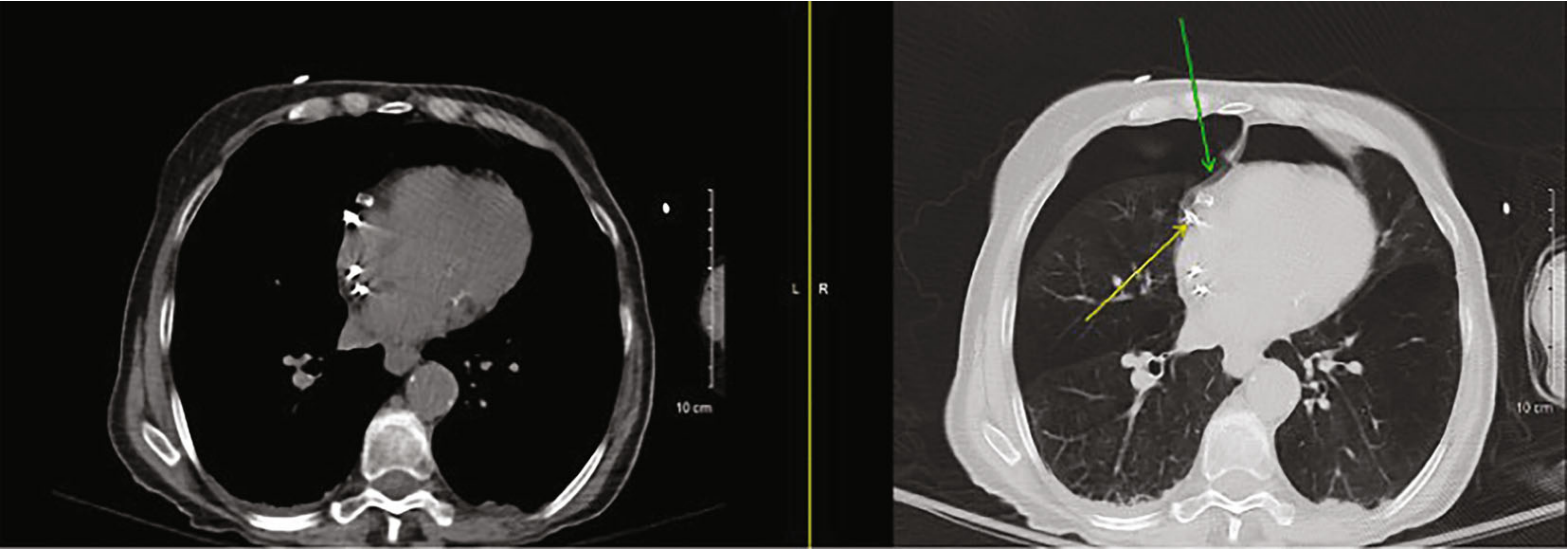
Yellow arrow showing the RA lead penetrating the atrial wall and green arrow showing the pneumopericardium.

**Figure 3:**
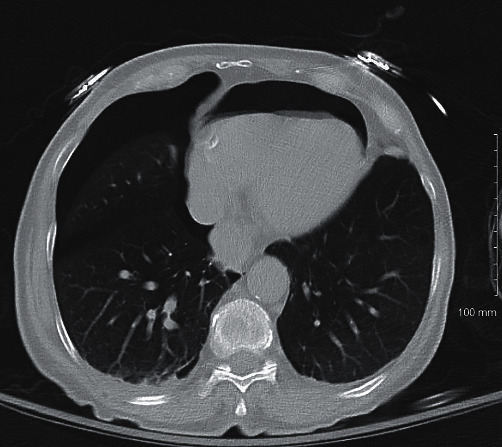
Noncontrast chest CT scan showing pneumopericardium, pneumothorax, and pericardial effusion.

**Figure 4:**
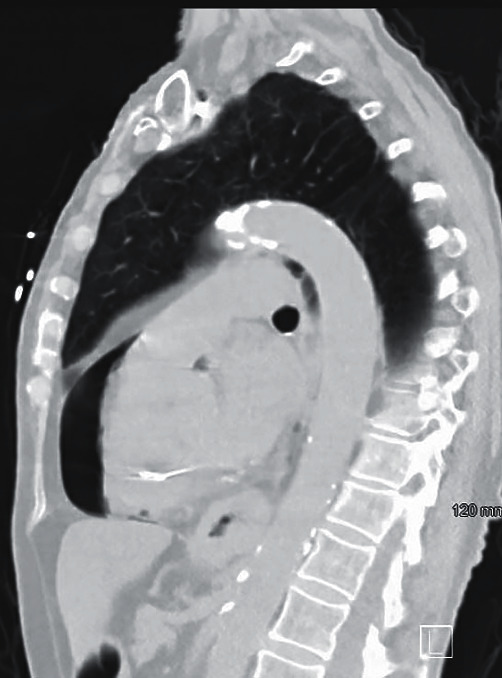
CT chest sagittal view demonstrating pneumopericardium.

**Figure 5:**
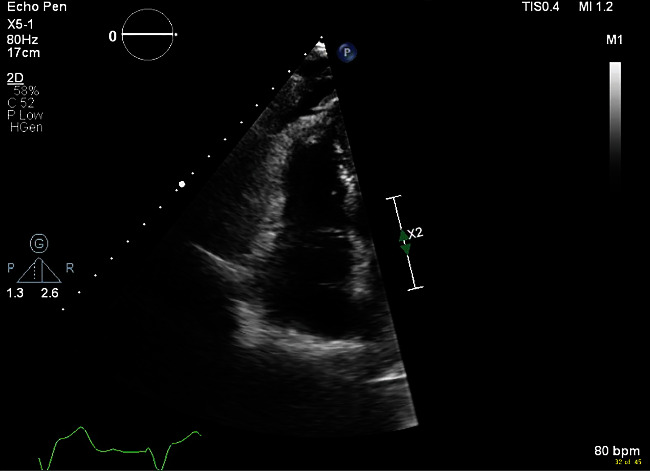
Transthoracic echocardiogram revealing pneumopericardium.

The initial chest X-ray after implantation showed right lower lobe anterior pneumothorax and pneumopericardium (Figure 1). A computerized tomography (CT) scan of the chest was done to investigate the etiology of the contralateral pneumothorax that revealed right atrial lead projecting through the right atrial wall and right ventricular lead at the wall (Figure 2). There was moderate right pneumothorax and small right effusion and moderate pneumopericardium anteriorly, about 2.3 cm thick. Small amount of air was also visualized anterior and posterior to aorta likely mild pneumomediastinum (Figures 2–4). Transthoracic echocardiogram was also repeated after the procedure that revealed pneumopericardium and normal right ventricular function (Figure 5).

Cardiothoracic surgery was consulted, and a 14 Fr pigtail chest tube was inserted in the sixth intercostal space. Device interrogation demonstrated no change in pacing threshold, sensing, normal impedance of both leads, and no issues with the functionality of pacemaker; therefore, right atrial (RA) lead repositioning was not required. Chest X-ray was repeated the next morning, following chest tube placement that revealed significant improvement in pneumothorax and pneumopericardium. A CT of the chest was repeated 4 days later and revealed complete resolution of the pneumothorax, pneumopericardium, and pneumopericardium. The patient was discharged home safely and had been symptom free for a 1-month follow-up.

## 2. Discussion

Despite a relatively low rate of complications ranging from less than 1 to 6% with cardiac pacemaker implantation, it is pertinent to early recognition and timely management of these complications [2]. The most common complications include risk of bleeding, phlebitis, lead dislodgement, infection, hemothorax, pneumothorax, pneumopericardium, and even cardiac perforation [3].

The rate of some specific early complications like subclavian vein rupture and pneumothorax has been found to be higher with dual chambers when compared to single chamber devices [4].

Chest X-ray, echocardiogram, and device interrogation are all helpful modalities in determining immediate post-implantation complications. A CT of the chest [3] has the highest sensitivity in revealing all complications associated with pacemaker implantation including pneumomediastinum.

Commonly, pneumothorax is a complication visualized on the ipsilateral side of Permanent pacemaker (PPM) insertion; however, in rare instances, contralateral pneumothorax can also develop as seen in this case. Possible mechanisms for contralateral pneumothorax include the injury to pleura by the RA lead extrusion through the atrial appendage or pleural injury by guide-wire during Seldinger set introduction.

Development of pneumopericardium and pneumomediastinum on the contralateral side of PPM placement is likely due to perforation of the atrial lead through the wall of the atrial appendage. Penetration of the RA lead does not change lead parameters due to penetration of the helix of the lead, which is not part of the electrode, and the electrode remains in contact with the myocardium. The lead may plug the micro-defect and, therefore, no pericardial effusion [5].

Management of such rare complications after pacemaker implantation depends on the patient's hemodynamic status, device interrogation, and amount of air and blood leak.

Srivathsan et al. performed both chest tube insertion and atrial lead extraction and had a successful outcome [6]. At the same time, in other cases of similar complications, chest tube insertion without right atrial lead extraction management resulted in resolution of both pneumothorax and pneumopericardium [5, 7].

## Data Availability

The data used to support the findings of this study are included within the article.
